# Supplementary material for: Identification and Characterization of an RRM-Containing, RNA Binding Protein in Acinetobacter baumannii
Source: Biomolecules. 2022 Jun 30;12(7):922. doi: 10.3390/biom12070922 (PMC9313427; doi:10.3390/biom12070922)
Supplement: Supplementary file 1 [file biomolecules-12-00922-s001.zip › Supplementary Figures.pdf]

Supplementary figures of

Identification and characterization of an RRM-containing, RNA binding protein in *Acinetobacter Baumannii*

Caterina Ciani[1], Anna Pérez-Ràfols[2], Isabelle Bonomo[1], Mariachiara Micaelli[1], Alfonso Esposito[3], Chiara Zucal[1], Romina Belli[4], Vito Giuseppe D'Agostino[1], Irene Bianconi[1], Vito Calderone[2], Linda Cerofolini[2], Orietta Massidda[1], Michael Bernard Whalen[5], Marco Fragai[2]* and Alessandro Provenzani[1]*

[1] Department of Cellular, Computational and Integrative Biology, DeCIBIO, University of Trento, Trento, Italy
[2] Magnetic Resonance Center (CERM) – Department of Chemistry "Ugo Schiff", University of Florence, and Consorzio Interuniversitario Risonanze Magnetiche di Metalloproteine (CIRMMP) and Giotto Biotech, S.R.L, Florence, Italy
[3] International Centre for Genetic Engineering and Biotechnology, (ICGEB), Padriciano, 99, Trieste, 34149 Italy
[4] Proteomics and MS Core Facility, Department of Cellular, Computational and Integrative Biology, DeCIBIO, University of Trento, Trento, Italy
5 Institute of Biophysics (IBF), National Research Council (CNR), FBK Nord, Trento, Italy
* Correspondence: alessandro.provenzani@unitn.it; fragai@cerm.unifi.it

Figure S1 1

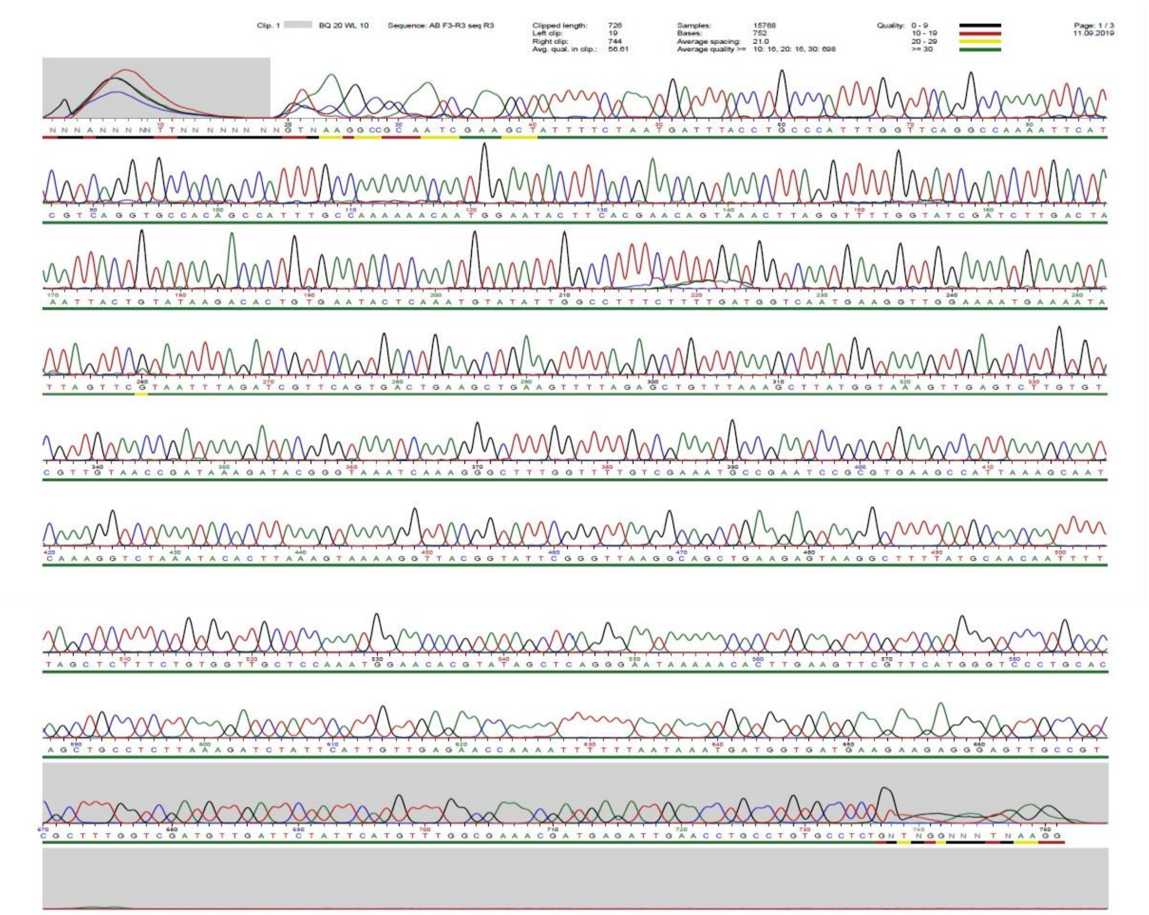

1) Sanger sequence of the retro-transcribed amplicon of the transcriptome of Acinetobacter baumannii. We used the reverse primer R3 that was designed 235 nucleotides downstream the gene of interest. This data confirmed the insertion of the mRNA sequence into a bigger polycistronic mRNA and confirmed the possible expression of the protein AB-Elavl.

2

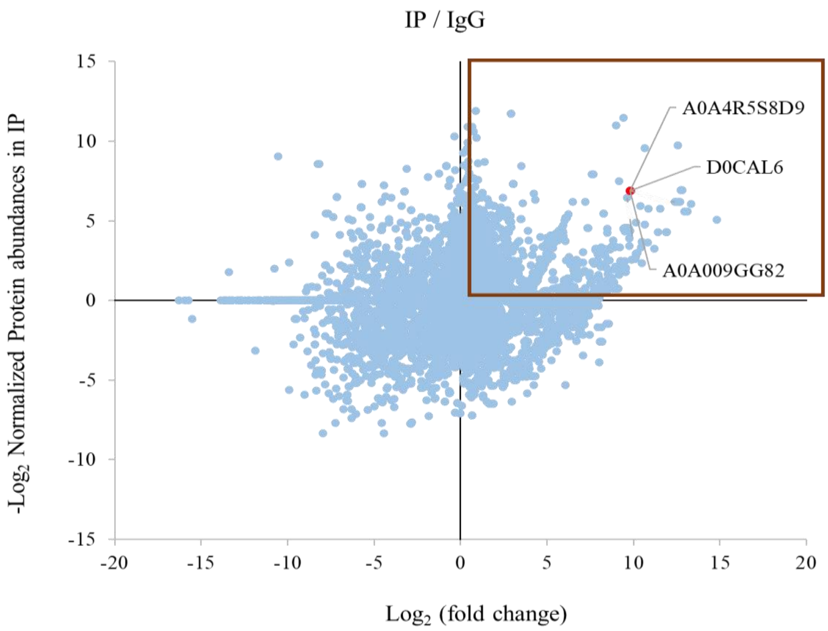

2) Scatter plot comparing protein enrichment (log2 FC IP-vs-IgG) compared with protein abundance within the IP proteome. Hypothetical and highly similar RNA binding proteins of *A. baumannii* are highlighted.

3

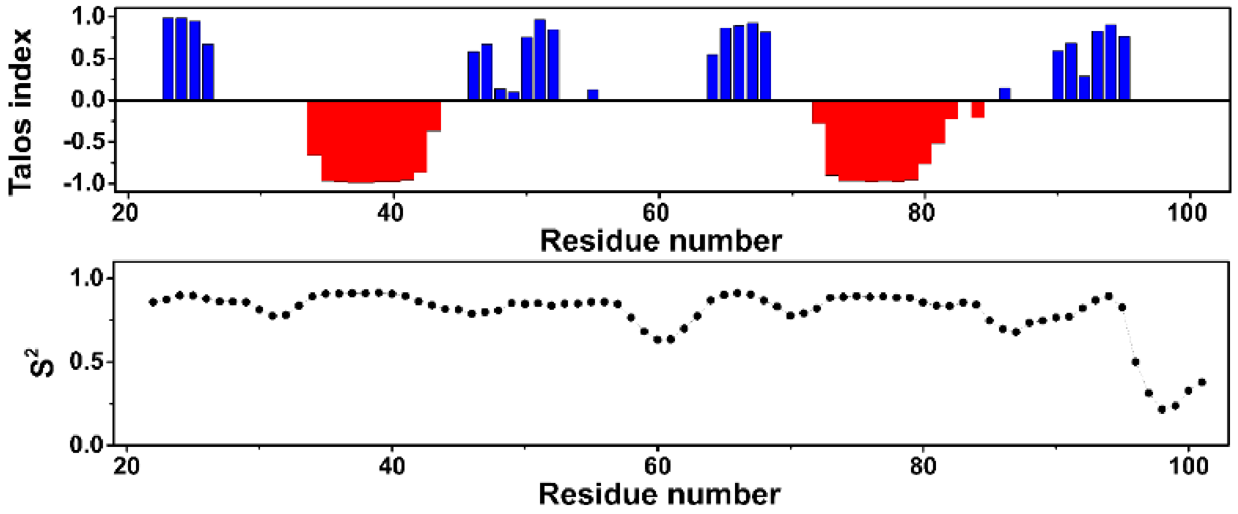

3) Top, secondary structure prediction obtained by the program Talos+ using the experimental values of chemical shifts of HN, N, C′, Cα, and Cβ atoms as input data. The blue bars indicate the β-strand propensity while the red bars the α-helix propensity. Bottom, predicted order parameter (S2) by the program Talos+ using the experimental values of chemical shifts of HN, N, C′, Cα, and Cβ atoms as input data.

4

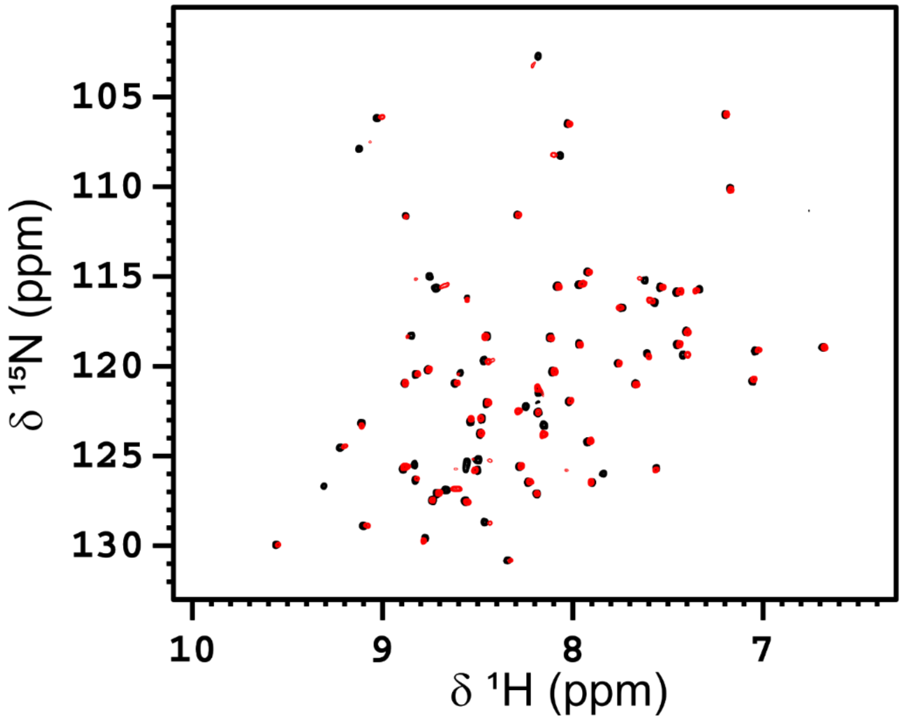

4) NMR analysis of the protein binding toward AREs. Superimposed 2D 1H-15N HSQC spectra of free AB-Elavl RRM domain (70 µM, black) and in the presence of 140 µM of ARE Pos (red). The spectra were acquired on a spectrometer operating at 950 MHz and 298 K.

5

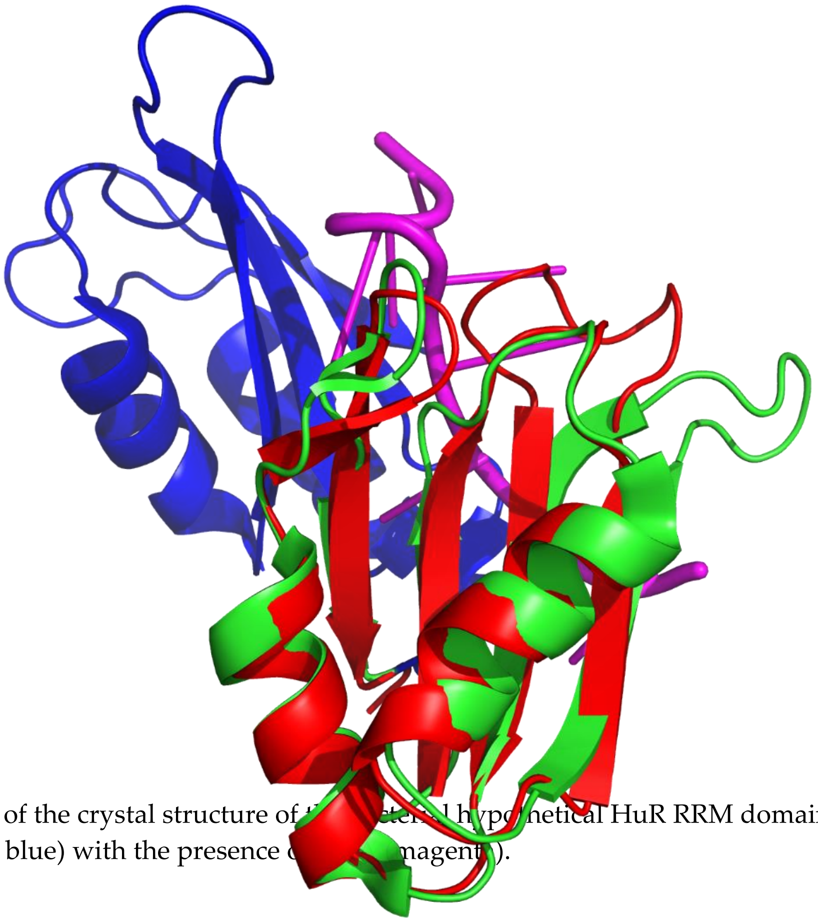

5) Superposition of the crystal structure of [illegible] hypothetical HuR RRM domain (rAB-Elavl, red) and 1FLX (green and blue) with the presence [illegible] magent[illegible]).

6

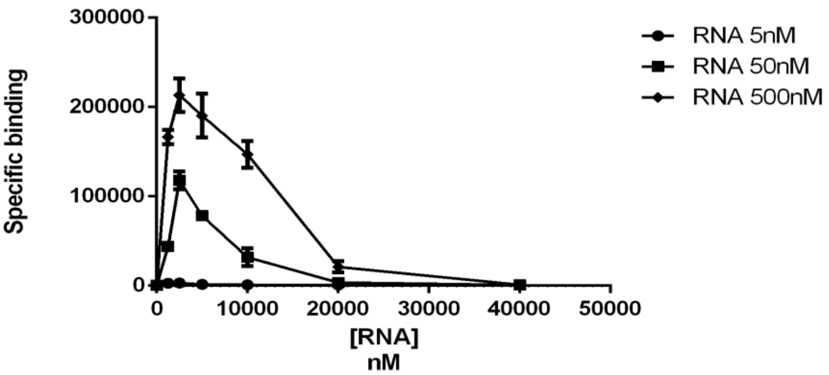

6) AlphaScreen for detection of the hook point on the recombinant protein and three different concentrations of AREpos probe, to have the best signal/noise ratio. [protein]: 250nM; [RNA]: 50nM.

7

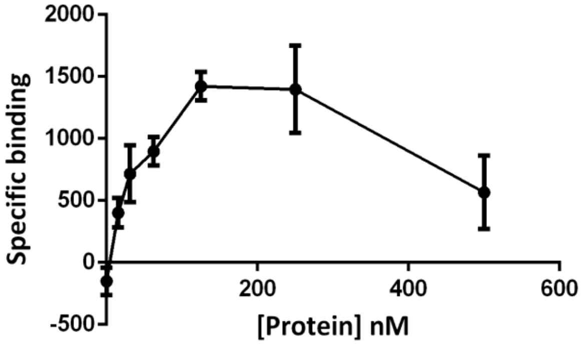

7) Hook point established by HTRF-FRET between the recombinant protein and AREpos.
