# Supplementary material for: Identification and Characterization of an RRM-Containing, RNA Binding Protein in Acinetobacter baumannii
Source: Biomolecules. 2022 Jun 30;12(7):922. doi: 10.3390/biom12070922 (PMC9313427; doi:10.3390/biom12070922)
Supplement: Supplementary file 1 [file biomolecules-12-00922-s001.zip › Supplementary Table S2.pdf]

Supplemetary Table S2

Fasta format entry F3P16_RS16475 RNA-binding protein [Acinetobacter baumannii]

Gene ID: 66398575, updated on 15-Nov-2021

Gene symbol F3P16_RS16475

>NZ_CP043953.1:c3494438-3494199 Acinetobacter baumannii strain K09-14 chromosome, complete genome

ATGAAAATATTAGTTCGTAATTTAGATCGTTCAGTGACTGAAGCTGAAGTTTTAGAGCTGTTTAAAGCTT

ATGGTAAAGTTGAGTCTTGTGTCGTTGTAACTGATAAAGATACGGGTAAATCAAAGGGCTTTGGTTTTGT

CGAAATGCCGAATCCGCGTGAAGCCATTAAAGCAATCAAAGGTCTAAATACACTTAAAGTAAAAGGTTAC

GGTATTCGGGTTAAGGCAGCTGAAGAGTAA

Predicted protein sequence

> WP_000699342.1

1 mkilvrnldr svteaevlel fkaygkvesc vvvtdkdtgk skgfgfvemp npreaikaik

61 glntlkvkgy girvkaaee
